# DNA methylation of claudin-6 promotes breast cancer cell migration and invasion by recruiting MeCP2 and deacetylating H3Ac and H4Ac

**DOI:** 10.1186/s13046-016-0396-x

**Published:** 2016-07-26

**Authors:** Yafang Liu, Xiangshu Jin, Yanru Li, Yang Ruan, Yan Lu, Minlan Yang, Dongjing Lin, Peiye Song, Yantong Guo, Shuai Zhao, Bing Dong, Yinping Xie, Qihua Dang, Chengshi Quan

**Affiliations:** 1 The Key Laboratory of Pathology, Ministry of Education, College of Basic Medical Science, Jilin University, 126 Xinmin Street, Changchun, Jilin 130021 China; 2 Department of Pathology, the First Affiliated Hospital of Jilin University, 126 Xinmin Street, Changchun, Jilin 130021 China

**Keywords:** Tight junction, Breast cancer, CLDN6, Methylation

## Abstract

**Background:**

Claudin-6 (CLDN6), a member of claudin transmembrane protein family, has recently been reported to be undetectable or at low levels in human breast cancer cell lines and tissues and plays a role in suppression of migration and invasion in breast cancer cells. In addition, it is reported that CLDN6 expression is regulated by DNA methylation in various human cancers and cell lines. However, it is unclear how DNA methylation regulates CLDN6 expression. Here we show the mechanism by which DNA methylation regulates CLDN6 expression in human breast cancer cell line MCF-7.

**Methods:**

RT-PCR, Western blot and immunofluorescent staining were utilized to investigate CLDN6 expression in breast cancer tissues and MCF-7 cells. Methylation-Specific PCR (MSP) was applied to determine DNA methylation status in CLDN6 gene promoter region. Wound-healing assay and invasion assay were utilized to test mobility of MCF-7 cells treated with 5-aza-dC (DNA methyltransferase inhibitor). MeCP2 binding, H3Ac and H4Ac in CLDN6 promoter region were analyzed by ChIP assay. Nuclease accessibility assay was performed for analysis of the chromatin conformation of CLDN6 gene. To study the role of CLDN6 in malignant progression, we used RNAi to knockdown CLDN6 expression in MCF-7 cells treated with 5-aza-dC, and examined the mobility of MCF-7 cells by wound-healing assay and invasion assay.

**Results:**

5-aza-dC and TSA (histone deacetylase inhibitor) application induced CLDN6 expression in MCF-7 cells respectively and synergistically. 5-aza-dC treatment induced CLDN6 demethylation, inhibited MeCP2 binding to CLDN6 promoter and increased H3Ac and H4Ac in the promoter. In addition, TSA increased H4Ac, not H3Ac in the promoter. The chromatin structure of CLDN6 gene became looser than the control group after treating with 5-aza-dC in MCF-7 cells. 5-aza-dC up-regulated CLDN6 expression and suppressed migration and invasion in MCF-7 cells, whereas CLDN6 silence restored tumor malignance in MCF-7 cells.

**Conclusions:**

DNA methylation down-regulates CLDN6 expression through MeCP2 binding to the CLDN6 promoter, deacetylating H3 and H4, and altering chromatin structure, consequently promoting migratory and invasive phenotype in MCF-7 cells.

## Background

Claudins are structurally and functionally crucial components of tight junctions, which play important roles in regulating paracellular permeability and maintaining cell polarity in epithelial and endothelial cell sheets. Abnormal breast epithelial cell polarity causes epithelial-mesenchymal transition (EMT), and promotes tumor metastasis and invasion [1, 2]. The CLDNs family consists of 27 members, which are expressed in a tissue- and cell-specific manner [3, 4]. Loss of CLDNs expression has also been reported in several malignancies, e.g. loss of claudin 1 expression in breast cancer and in head and neck cancer.

In the present study, the expression of CLDN6 was undetectable or at low levels in human and rat mammary cancer cell lines and tissues [5]. Besides, tissues derived from breast carcinoma with lymph node metastasis were also accompanied with decreasing expression of CLDN6.

It is reported that the CLDN6 over-expression could inhibit migratory and invasive abilities in MCF-7 cells with stable CLDN6 expression [6]. Similarly, CLDN6 expression may suppress breast tumorigenesis [7]. These results revealed that CLDN6 over-expression might contribute to suppression of breast cancer progression. Nevertheless, the mechanism for aberrant CLDNs expression has not been explored yet. Silence of tumor suppressor gene may relate to the epigenetic events. Cancer epigenetic events such as aberrant DNA methylation and histone modification lead to inactivation of specific tumor-suppressor genes [8, 9]. DNA methylation is an important epigenetic modification that affects chromatin structure and gene expression [10]. Tumor suppressor gene silence is involved in carcinogenesis, drug resistance and recurrence [11–13]. High levels of methylation were associated with disease recurrence. Particularly, MLH1, ATM and FHIT gene promoters are found to be significantly hypermethylated in recurring adenomas [14]. DNA methylation leads to transcriptional repression either by methyl groups which interfere with protein binding to its cognate DNA sequence or by recruiting proteins that specifically recognize methylated CpGs (mCpGs) [15], the prototype of which is methyl-CpG-binding protein 2 (MeCP2). In the enzymatic mode, MeCP2 specifically targets mCpGs and determines repressive heterochromatin by interacting with co-repressor mSin3A, which is a part of a large complex containing histone deacetylases (HDACs) [16, 17]. MeCP2 can also recruit histone methyltransferase that methylates local H3 lysine9 for strengthening the repressive state [17].

In our study we firstly examined CLDN6 expression and methylation status at CLDN6 gene CpG sites in breast cancer tissues and MCF-7 cells to study relationship between CLDN6 expression and DNA methylation. To explore the molecular mechanism of DNA methylation of CLDN6, we investigated the relevance among CLDN6, MeCP2, H3Ac and H4Ac in MCF-7 cells. Our investigation showed that CLDN6 expression was associated with DNA methylation in breast cancer tissues and cells. Moreover, DNA methylation of CLDN6 promoter down-regulated its expression through binding with MeCP2, deacetylating H3 and H4, and altering chromatin structure, ultimately resulted in malignant phenotype in MCF-7 cells. In addition, we demonstrated that DNA methyltransferase inhibitor 5-aza-dC and histone deacetylase inhibitor TSA treatment induced CLDN6 expression respectively and synergistically, and suppressed migration and invasion in MCF-7 cells. However, knockdown CLDN6 expression with 5-aza-dC treatment would enhance the invasive and migratory abilities.

## Methods

### Patients’ samples and cell culture

Paraffin-embedded tissues from 30 specimens of breast cancer including 19 specimens with lymph node metastasis, 11 specimens without lymph node metastasis and 10 specimens of breast pericarcinomatous tissues were collected from patients at the Third Hospital of Jilin University with approval by the institutional review board (IRB). None of the patients received neo-adjuvant therapy. The patients’ medical records were reviewed to obtain their age, tumor status and clinical stage. All the cancer cases were classified and graded according to the International Union Against Cancer (UICC) staging system for breast cancer.

Human breast epithelial cell line HBL-100, human breast adenocarcinoma cell line MCF-7 and ovarian cancer cell line COC1 were cultured in Dulbecco’s modified Eagle’s medium (DMEM) (GIBCO, USA) supplemented with 10 % fetal bovine serum (FBS) (BD, Tokyo, Japan) at 37 °C in a humidified 5 % CO_2_ atmosphere.

### Reverse transcription—PCR (RT- PCR)

Total RNA was isolated using TRIzol reagent (Invitrogen, USA). The concentration of RNA was measured by the absorbance at 260 nm. 0.5 μg total RNA was reverse transcribed using M-MLV reverse transcriptase (TaKaRa, Japan) and random primer (TaKaRa, Japan) at 42 °C for 60 min and 1 μl of the final cDNA reaction was used for subsequent PCR reaction using Taq DNA polymerase (TaKaRa, Japan). Amplification conditions were as follows: PCR reactions were denatured at 94 °C for 2 min, followed by 28 cycles of 94 °C for 30s, 50–60 °C for 30s, and 72 °C for 1 min. This was followed by a final extension of 10 min at 72 °C. β-actin and GAPDH were used as endogenous control.

The PCR primers were summarized in Table 1. The reaction products were resolved on 1.5 % agarose gels and visualized by staining with ethidium bromide, the image was observed and photographed under viltalight lamp using Gel Imaging System (Bio-Rad Laboratories Inc, Hercules, CA, USA). The results were analyzed by Quantity One 4.4.1 software (Bio-Rad Laboratories Inc, Hercules, CA, USA).

**Table 1:** Primers for PCR assay

Gene name	Sequence (5’ → 3’)
Reverse transcription PCR primers	
CLDN6	
Sense	CACTGCCACTTCTGGATGG
Antisense	CAGTGCAGCTCCTTCAACCT
β-actin	
Sense	CCACTGCGTCGCGGGG
Antisense	GGCAGCCAGCTCAGCCATG
GAPDH	
Sense	TGTTGCCATCAATGACCCCTT
Antisense	CTCCACGACGTACTCAGCG
Methylation-Specific PCR primers	
Unmethylated DNA	
Sense	TGGATGTTTGTTAGTTTGAGGT
Antisense	ATAACCACAACCCAAATTCACA
Methylated DNA	
Sense	ACGTTTGTTAGTTCGAGGC
Antisense	ATAACCGCAACCCGAATTC
ChIP primers	
CLDN6	
Sense	CCCATCCCCACGCACGCTT
Antisense	GCCACCTGGATGGGCGAGTC
Nuclease Accessibility primers	
Primer1	
Sense	TCTCCCCAAGGTGGTCTGCT
Antisense	CCTTTTCAAATCCAGGTTCGGC
Primer2	
Sense	GGCGGTGGTCCAGTGACGT
Antisense	TAAAGGGAATAG AGGAAGCGTCC
Primer3	
Sense	AGTCTCCCCAAGGTGGTCTGC
Antisense	CCTTTTCAAATCCAGGTTCGGC
Primer4	
Sense	AGGAATTTCCCTTATCTCCTTCGC
Antisense	CCCATCCCCACGCACGCTT
Primer5	
Sense	GCCACCTGGATGGGCGAGTC
Antisense	GAGGGTTCCCAATTTGCGGG
Primer6	
Sense	CAGGGACAA AGACTCCGAAACC
Antisense	CTGGAACGCCGGTGACGT
Primer7	
Sense	CTCCCCAAGGTGGTCTGCT
Antisense	TCAAATCCAGGTTCGGCATGTTA
Primer8	
Sense	CCACTGCAGCTATAGAACCCCTCCA
Antisense	TTCTTTCCCCGTCCCCG

### Western blot

Total protein was extracted using 400 μl protein extraction fluid (HuaTe Sheng, China) with 40 μl (10 mM) phenylmethanesulfonyl fluoride (PMSF) according to the supplier’s instructions. The concentration of the total protein was determined using BCA Protein Assay Kit (Pierce Chemical Co, USA). 60 μg of denatured total protein of each sample per lane were resolved by SDS-PAGE (15 % acrylamide), and transferred to a nitrocellulose membrane (Millipore, USA) using a Semi-DRY Transfer cell (Bio-Rad Laboratories Inc, Hercules, CA, USA). The membrane was blocked with 5 % defatted milk (in 25 mM Tris, pH 8.0, 125 mM NaCl, 0.1 % Tween 20) for 1 h at room temperature then incubated with 1:1000 diluted anti-CLDN6 (Santa Cruz Biotechnologies) antibody at 4 °C overnight. After washing three times with PBS, the membrane was incubated with a horseradish peroxidase conjugated anti-goat IgG (1:1000, Santa Cruz Biotechnologies) secondary antibody at room temperature for 1 h. After washing, the immunoreactive bands were visualized using an ECL western blot system (GE, USA) and exposed to X-ray film (Estman Kodak). Anti-β-actin (Santa Cruz Biotechnologies) antibody was used as the endogenous control.

### Methylation-specific PCR analysis

Genomic DNA was extracted from MCF-7 cells using Wizard Genomic DNA Purification Kit (Promega), and 1 μg DNA was denatured by 0.3 M NaOH for 10 min at 37 °C. All samples were incubated at 50 °C for 16 h after adding hydroquinone (Sigma, USA) and sodium bisulfate (Sigma, USA). Methylation-specific PCR (MSP) was performed to analyze the methylation status at CpG-rich area of CLDN6 promoter. Primers specific for unmethylated DNA and methylated DNA were summarized in Table 1. PCR was carried out for 30 cycles at 95 °C for 30s, 58 °C for 30s, followed by a final extension at 72 °C for 10 min. PCR products were electrophoresed on 1.5 % agarose gel stained with ethidium bromide.

### 5-Aza-2’-deoxycytidine (5-aza-dC) and Trichostatin A (TSA) Treatment

MCF-7 cells were treated with various concentrations (0, 2.5, 5, 10, 15, and 20 μM) of DNA methyltransferase inhibitor, 5-aza-dC (Sigma, USA) for 24 h or 48 h, and various concentrations (0, 0.075, 0.125, 0.25, 0.5, and 1 μM) of histone deacetylase inhibitor, TSA (Sigma, USA) for 48 h respectively, and treated with 20 μM of 5-aza-dC with 0.125 μM TSA for 48 h.

### Immunofluorescent staining

MCF-7 cells were seeded onto small coverslips and treated with 17-β-estradiol at 0.005 μM for 24 h. After washing three times with PBS, the cells were fixed with 4 % paraformaldehyde for 30 min at room temperature. The cells were incubated with 0.3 % H_2_O_2_ and 30 μl goat serum 30 min respectively. The cells were incubated with 1:500 diluted anti-CLDN6 antibodies (Santa Cruz Biotechnologies) at 4 °C overnight and 1:50 diluted Rhodamine (red) -conjugated anti-goat IgG was used as a secondary antibody. The cells were photographed by confocal microscope (OLYMPUS, Japan).

### Wound-healing assay

Cancer cells were cultured in monolayer at 90 % confluence on gridded plastic dishes, and then wounded by scratching them with a 200 μl pipette tip, and then washed 3 times. The cells were cultured in Dulbecco’s modified Eagle’s medium (DMEM) (GIBCO, USA) supplemented with fetal bovine serum free (BD, Tokyo, Japan) at 37 °C in a humidified 5 % CO_2_ atmosphere. The wounds were photographed at 0 and 24 h.

### Invasion assay

The invasive potential of cancer cells was determined using Boyden chamber. MCF-7 cells were first cultured in DMEM with 10 % fetal bovine serum to 90 % confluence, and then washed 2 times with PBS. Then the cells were cultured in FBS free medium for 24 h at 37 °C in a humidified 5 % CO_2_ atmosphere. The medium containing chemotactic factors from the cell culture was collected. Matrigel was added to the upper boyden chamber of 24-well and incubated at 37 °C for 30 min. Then it was followed by adding medium with chemotactic factor to 24-well, putting MCF-7 cells suspension onto the matrigel and incubating at 37 °C in a humidified 5 % CO_2_ atmosphere 24 h. In the end, the invasive cells were counted.

### ChIP Assay

The chromatin immunoprecipitation was performed using the EZ Magna ChIP G chromatin immunoprecipitation kit (Millipore). Briefly, MCF-7 cells were treated with 20 μM 5-aza-dC. The protein-DNA complexes of cells were cross-linked using formaldehyde at room temperature for 10 min. The genomic DNA of lysed cells was sheared to 200–1000 bp by sonication. The final lysate was incubated with MeCP2-, and MBD2-specific antibodies and precipitated with protein G magnetic beads. After washing three times, DNA-protein complexes were reversely cross-linked, and genomic DNA was extracted with Wizard Genomic DNA Purification Kit (Promega, USA), and eluted in 50 μl of TE buffer. 0.5 μg DNA was used for PCR as described above. The PCR primers were summarized in Table 1. The products of PCR amplification were separated on 2.0 % agarose gel and visualized using ethidium bromide and UV light. The results were analyzed as described above.

### Quantitative RT-PCR

To quantify amplified signals from the CLDN6 5’ flanking region and promoter in MCF-7 cells, quantitative PCR was undertaken by using the iCycleriQ real-time PCR detection system (Bio-Rad, Hercules, CA, USA) and an iQ SYBR green master mix (Bio-Rad, Hercules, CA, USA). The PCR cycles were 95 °C for 2 min and then 40 cycles of 95 °C for 15 s, 60 °C for 30s. The primers were designed to amplify 100 bp per primer pair and summarized in Table 1. The specificity of amplification for each pair was confirmed by separation of amplified fragments by agarose electrophoresis and by performing melting curve analysis. PCR assays were run in triplicate.

### Nuclease accessibility assay

The nuclei of MCF-7 cells were prepared as described [18]. Nuclei (100 μg DNA) were treated with 500 units of nuclease S7 (TaKaRa, Japan) for 10 min at 37 °C to produce mononucleosomal fragments. The mixture was treated with proteinase K, and DNA was purified using phenolchloroform. The digested product was electrophoresed on 1.2 % agarose gel stained with ethidium bromide. To analyze the extent to which different parts of the CLDN6 promoter region were digested by S7 nuclease, we used real-time PCR as described above. PCR assays were run in triplicate and the relative abundance of CLDN6 promoter regions was estimated from the threshold amplification cycle number, Ct, using software supplied with iCycler IQ. The differences between Ct values obtained from MCF-7 cells treated with 20 μM 5-aza-dC and control cells were plotted against corresponding primer pairs that amplified small, overlapping sequences within the 5’flanking region of the CLDN6 gene (primer sequences used in this assay are available upon request). The primers were summarized in Table 1.

### Short hairpin RNA (shRNA) transfection

Four shRNAs were designed, based on the claudin-6 mRNA sequence NM_021195 and were constructed into the vector pGCsilencer™U6/Neo/GFP (GeneChem, Shanghai, China). MCF-7 cells were transfected with shRNA-CLDN6 using SuperFect Transfection Reagent (QIAGEN, USA). A negative control cell line was generated by transfecting cells with the vector constructed by targeting a sequence that did not yield any appreciable knockdown of the protein production. shRNA sequences were as follows: i) caGTCAAGCTATGGAACTAATTTCAAGAGAATTAGTTCCATCATAGCTTGACtg; ii) aaCTGAGCCAAGGTGTTGACTTTCAAGAGAAGTCAACACCTTGGCTCAGtt; iii) gcCAGATGCAGTGCAAGGTGTTTCAAGAGAACACCTTGCAACTGCATCTGgc and iv) caGTGCAAGGTGTACGACTCATTCAAGAGATGAGTCGTACACCTTGCACtg. shRNA-mediated suppression of CLDN6 expression was confirmed using RT-PCR and Western blot as described above.

### Statistical analysis

Counts and rates were calculated for categorical variables. The Chi-square test was used to compare categorical variables among different groups. All statistical analyses were performed using the SPSS software package (version 13.0.0). *P* values less than 0.05 were considered statistically significant.

## Results

### CLDN6 expression is down-regulated in breast cancer tissues and MCF-7 cells, correlating with DNA methylation

**Fig. 1:**
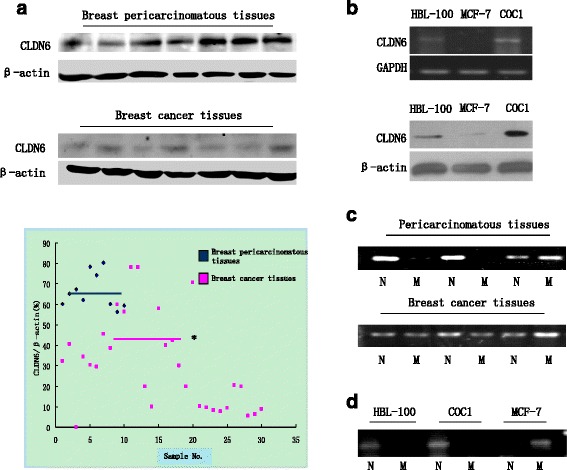
CLDN6 expression is down-regulated drastically by DNA hypermethylation in breast cancer tissues and MCF-7 cells. **a** CLDN6 expression was significantly higher in breast pericarcinomatous tissues than that in breast cancer tissues by western blot. From lane 1 to lane 7 were represented of different tissue samples. *P* = 0.021 < 0.05. **b** The expression of CLDN6 in HBL-100、MCF-7 and COC1 cells (as tissue-specific control), determined by RT-PCR and Western blot assay. Differently with HBL-100 cells, CLDN6 expression was undetectable or low levels in MCF-7 cells. **c** CLDN6 DNA methylation status was determined in breast pericarcinomatous tissues and breast cancer tissues by MSP. (D) CLDN6 DNA methylation status was determined by MSP analysis in HBL-100, MCF-7 and COC1 cells. CLDN6, claudin-6; MSP, Methylation-Specific PCR

The CLDN6 protein level was higher in breast pericarcinomatous tissues compared to breast cancer tissues (Fig. 1a). CLDN6 expression level is lower in the cancer samples with lymph node metastasis than in those without lymph node metastasis (Table 2). In addition, CLDN6 expression was inversely correlated with breast cancer cell metastasis, indicating that CLDN6 might play an important role in suppressing breast cancer progression.

**Table 2:** Clinical pathological characteristic of breast cancer patients associating with CLDN6 expression

Characteristic	*N*	CLDN6 Expression		*P* value
		High	Low	
Breast cancer tissues	30	7 (23.33 %)	23 (76.67 %)	
Pericarcinomatous tissues	10	10 (100 %)	0 (0 %)	*P* < 0.05
Age (years)				
20–40	9	2 (22.22 %)	7 (77.78 %)	
40–60	14	3 (21.43 %)	11 (78.57 %)	
60–80	7	2 (28.57 %)	5 (71.43 %)	*P >* 0.05
Tumor status				
T1	13	2 (15.38 %)	11 (84.62 %)	
T2	12	4 (33.33 %)	8 (66.67 %)	
T3	5	1 (20 %)	4 (80 %)	*P >* 0.05
Clinical stage				
Is-I	14	4 (28.57 %)	10 (71.43 %)	
II-IV	16	3 (18.75 %)	13 (81.25 %)	*P >* 0.05
Node status				
+	19	2 (10.53 %)	17 (89.47 %)	
–	11	5 (45.45 %)	6 (54.55 %)	*P* < 0.05

*P* value was measured with student’s t test. Significance of association was determined using a χ^2^ test

The comparison of mRNA and protein level of CLDN6 expression among breast cancer MCF-7 cells, HBL-100 cells (human normal breast cells) and COC1cells (human cervical cancer cells as tissue-specific control) showed that CLDN6 expression was decreased in MCF-7 as compared with HBL-100 and COC1, indicating that reduction of CLDN6 expression had tissue- and cell-specificity, which was consistent with clinical assays (Fig. 1b).

Aberrant DNA methylation such as localized CpG island hypermethylation leads to inactivation of specific tumor-suppressor genes [19–21]. To investigate CpG island distribution, we used Methyl Primer Express software to search CpG islands in CLDN6 promoter region and found CpG islands were enriched in the region. Then we hypothesized that the low expression of CLDN6 might be correlated with DNA methylation in breast cancer. Figure 1c showed DNA methylation of CLDN6 in breast pericarcinomatous tissues and cancer tissues by MSP assay respectively. 20 % of 10 breast pericarcinomatous tissues showed CLDN6 methylation, while 60 % of 30 breast cancer tissues showed CLDN6 methylation (Table 3). In addition, 69.9 % of 23 breast cancer tissues showed DNA methylation with low expression of CLDN6, indicating that CLDN6 expression was negatively associated with DNA methylation (Table 4). Figure 1d showed that CLDN6 CpG sites were hypermethylated in MCF-7 cells. The results suggested that dramatically down-regulated CLDN6 expression may be correlated with DNA methylation of CLDN6.

**Table 3:** DNA methylation of CLDN6 in breast pericarcinomatous tissues and cancer tissues

Type of tissues	Methylation		Positive rate
	–	+	
Breast cancer	12/30	18/30	60 %
Pericarcinomatous tissues	8/10	2/10	20 %

χ^2^ = 8.27,*P* = 0.0268 < 0.05

**Table 4:** DNA methylation associating with CLDN6 expression in breast pericarcinomatous tissues and cancer tissues

CLDN6 expression	DNA Methylation		*P* value
	-	+	
Low	7/23	16/23	*P* < 0.05
High	13/17	4/17	

Pearson’s chi-square (χ^2^) test,*P* = 0.033 < 0.05

### 5-aza-dC application induces CLDN6 expression and inhibits migratory and invasive abilities in MCF-7 cells

**Fig. 2:**
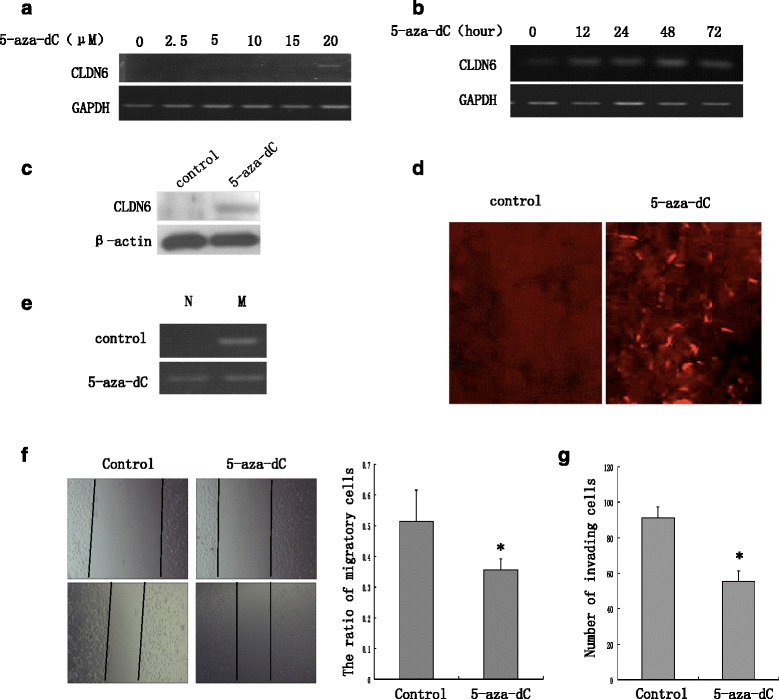
5-aza-dC application induces CLDN6 expression and inhibits migration and invasion abilities in MCF-7 cells. **a** RT-PCR was used to display the effect of 5-aza-dC on CLDN6 expression. 5-aza-dC application induced CLDN6 expression in a concentration- and time-dependent manner. Lane 1 was control group; lanes 2 to 6 were treated with 5-aza-dC group (2: 2.5 μM; 3: 5 μM; 4: 10 μM; 5: 15 μM; 6: 20 μM). Data in lane 6 showed that 20 μM 5-aza-dC treatment had the greatest effect on inducing CLDN6 expression. **b** CLDN6 expression was determined in MCF-7 cells treated with 20 μM 5-aza-dC for 1 to 72 h. Lane 1 was control group, lane 2 to 5 were 20 μM 5-aza-dC treated group (2: 12 h; 3: 24 h; 4: 48 h; 5: 72 h). Data in lane 5 showed that 20 μM 5-aza-dC for 48 h had the greatest effect on inducing CLDN6 expression. **c** CLDN6 protein expression was significantly up-regulated in MCF-7 cells treated with 5-aza-dC at 20 μM for 48 h by western blot. **d** Immunofluorescent staining showed CLDN6 expression at the membranes of MCF-7 cells after treated with 20 μM 5-aza-dC for 48 h using × 100 magnification. **e** CLDN6 DNA methylation status was determined by MSP analysis between control group and treatment with 5-aza-dC 20 μM for 48 h group. 5-aza-dC treating group increased demethylation of CLDN6 CpG sites. **f** MCF-7 cells treated with 5-aza-dC were examined the ability of cell migration compared with control by wound-healing assay. **g** The invasive cells treated with 5-aza-dC were remarkably lower invasive ability than control group by invasion assay. *P < 0.05. CLDN6, claudin-6; 5-aza-dC, 5-Aza-2’-deoxycytidine; MSP, Methylation-Specific PCR

To investigate whether DNA hypermethylation could lead to reducing CLDN6 expression, we treated MCF-7 cells with DNA methyltransferase inhibitor 5-aza-dC [22]. Our results showed that treatment with 5-aza-dC increased CLDN6 mRNA expression in a dose- and time-dependent manner by promoting demethylation of the CLDN6 CpG sites. Treatment with 20 μM 5-aza-dC for 48 h increased CLDN6 mRNA and protein expression dramatically (Fig. 2a, b and c), and membranous expression of CLDN6 was detected by immunofluorescence (Fig. 2d). In addition, treatment with 20 μM 5-aza-dC for 48 h promoted demethylation of CLDN6 CpG sites (Fig. 2e). To evaluate whether demethylation of CLDN6 plays a role in breast cancer progression, we analyzed invasive and migratory abilities in MCF-7 cells treated with 5-aza-dC. Consistently with our hypothesis, DNA demethylation of CLDN6 indeed inhibited migration and invasion in MCF-7 cells (Fig. 2f and g).

### TSA application induces CLDN6 expression in MCF-7 cells

**Fig. 3:**
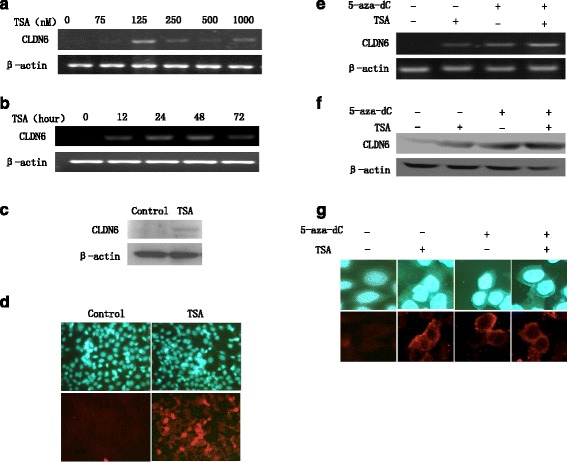
CLDN6 expression is down-regulated drastically correlating with histone deacetylization in breast cancer cells. **a** RT-PCR was determined effects of TSA treatment on CLDN6 expression in MCF-7 cells. TSA application induced CLDN6 expression in a concentration- and time-dependent manner. Lane 1 was control group, lanes 2 to 6 were TSA treated group (2: 75 nM; 3: 125 nM; 4: 250 nM; 5: 500 nM; 6: 1000 nM). Data in lane 3 showed that 125 nM TSA had the greatest effect on inducing expression of CLDN6. **b** CLDN6 expression in MCF-7 cells treated with 125 nM TSA for 1 to 72 h. Lane 1 was control group, lane 2 to 6 were 125 nM TSA treated group (2: 12 h; 3: 24 h; 4: 48 h; 5: 72 h). Data in lane 5 showed that 125 nM TSA for 48 h had the greatest effect on inducing expression of CLDN6. **c** Western blot assay examined CLDN6 protein expression was significantly up-regulated in MCF-7 cells treated with TSA at 125 nM for 48 h. **d** Immunofluorescent staining showed expression of CLDN6 at the membranes of MCF-7 cells treated with 125 nM TSA for 48 h using × 100 magnification. **e** RT-PCR was used to determine the expression of CLDN6 in MCF-7cells treated with 5-aza-dC and TSA respectively or synergistically. **f** Western blot assay examined CLDN6 protein expression in MCF-7cells treated with 5-aza-dC and TSA respectively or synergistically. **g** Immunofluorescent staining showed expression of CLDN6 at the membranes in MCF-7 cells treated with 5-aza-dC and TSA respectively or synergistically using × 400 magnification. CLDN6, claudin-6; TSA, Trichostatin A; 5-aza-dC, 5-Aza-2’-deoxycytidine

To investigate whether TSA plays a role in up-regulation of suppressor gene, we treated MCF-7 cells with TSA to investigate the involvement of histone deacetylation in CLDN6 gene silencing. After the treatment of MCF-7 cells with 125nM TSA for 48 h, mRNA and protein expression of CLDN6 were both increased (Fig. 3a, b and c), and membranous expression of CLDN6 was detected by immunofluorescence (Fig. 3d). Moreover, we found that simultaneous treatment of both 5-aza-dC and TSA enhanced CLDN6 expression synergistically (Fig. 3e, f and g). In summary, these results indicated that down-regulation of CLDN6 expression might be partially correlated with histone deacetylation in MCF-7 cells.

### Methylated DNA recruits MeCP2, and deacetylates H3Ac and H4Ac at CLDN6 promoter

**Fig. 4:**
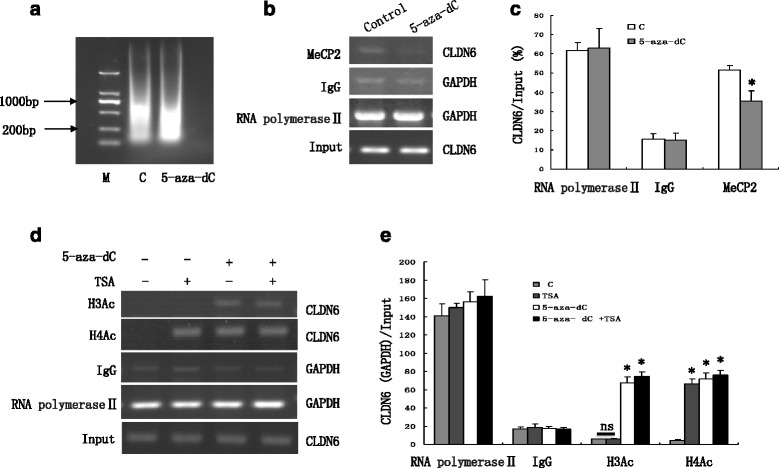
CLDN6 was down-regulated via recruiting MeCP2 and deacetylating H3Ac and H4Ac for DNA methylation stability. **a** The molecular size of shared genomic DNA of MCF-7 cells treated with DMSO (as control) and 5-aza-dC. **b** Effect of 5-aza-dC on binding of MeCP2 to CLDN6 was analyzed using ChIP assay. The binding of RNA polymerase II and IgG to the GAPDH promoter was used as a positive or a negative control. **c** Quantitative analysis of images presented in (**b**). **d** Analysis of CLDN6 promoter occupancy by H3Ac and H4Ac**.** ChIP was used to analyze the interactions of H3Ac and H4Ac with the CLDN6 proximal promoter in MCF-7 cells treated with 20 μM 5-aza-dC and 125nM TSA for 48 h respectively or synergistically. Effect of 5-aza-dC and TSA on binding of H3Ac and H4Ac to CLDN6 was analyzed using ChIP assay. The binding of RNA polymerase II and IgG to the GAPDH promoter was used as a positive and a negative control. **e** Quantitative analysis of images presented in (**d**). *P < 0.05. CLDN6, claudin-6; 5-aza-dC, 5-Aza-2’-deoxycytidine; TSA, Trichostatin A; MeCP2, methyl-CpG-binding protein 2; H3Ac, Histone 3 acetylation; H4Ac, Histone 4 acetylation

To further elucidate the mechanism of regulating CLDN6 methylation in breast cancer cells, we performed ChIP assay in MCF-7 cells treated with 5-aza-dC. MeCP2 binding to CLDN6 promoter was dramatically reduced in MCF-7 cells with 5-aza-dC treatment compared with those without the treatment (Fig. 4a, b and c). Moreover, 5-aza-dC application increased H4Ac and H3Ac in CLDN6 promoter region, but TSA application increased only H4Ac in the region. Besides, the combination treatment with 5-aza-dC and TSA increased H3Ac and H4Ac in CLDN6 promoter synergistically (Fig. 4d and e). Together, these results demonstrated that CLDN6 expression was down-regulated via recruiting MeCP2 and deacetylating H3Ac and H4Ac for stability of methylation.

### CLDN6 promoter is more accessible after micrococcal nuclease digestion in MCF-7 cells with 5-aza-dC treatment

**Fig. 5:**
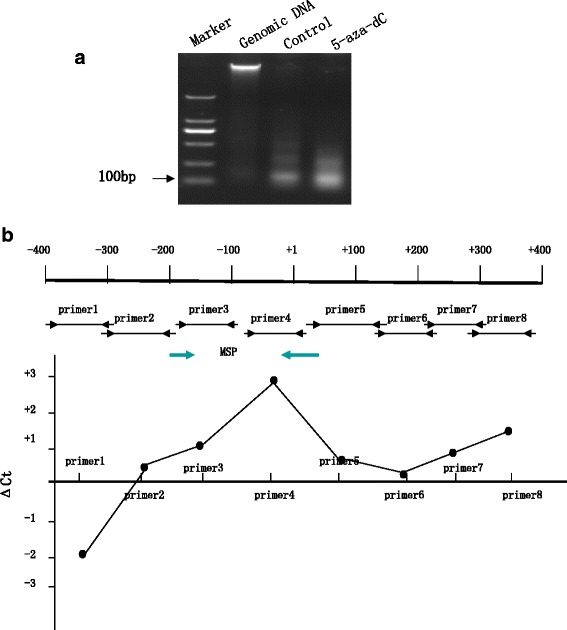
5-aza-dC may alter the chromatin structure of CLDN6 for transcription in MCF-7 cells. **a** MCF-7 cells were treated with micrococcal S7 nuclease to produce mononucleosomal genomic DNA fragments (100 ∼ 200 bp). **b** Primer pairs were designed between -400- + 400 bp of CLDN6 proximal promoter. Micrococcal S7 nuclease preferentially digested DNA that was not organized in nucleosomes. Hence, DNA organized in nucleosomes would be less prone to nuclease digestion and amplified to the qPCR threshold at a lower Ct compared to the less organized DNA. **c** Purified mononucleosomal DNA was amplified using primer pairs (depicted in (**b**)) and the resulting product was quantified using SYBR green dye in a quantitative PCR. The difference in Ct between 5-aza-dC treating MCF-7 and DMSO treating MCF-7 cells (as control) for each primer pair was plotted. The positive numbers suggested that in 5-aza-dC treating MCF-7 cells this part of the DNA was less organized into nucleosomes and more prone to nuclease digestion compared to control cells. CLDN6, Claudin-6; 5-aza-dC, 5-Aza-2’-deoxycytidine

To obtain information about nucleosomal organization of CLDN6 promoter, MCF-7 cells were treated with micrococcal S7 nuclease to digest internucleosomal DNA (Fig. 5a). Mononucleosomal DNA was isolated and amplified by utilizing overlapping and closely spaced (~100 bp) primer pairs. If the region to be amplified were more accessible by the nuclease in one cell type and less accessible in other cell types, we would detect their difference in the number of cycles required to reach the threshold level (Ct) in a quantitative PCR. Therefore, positive numbers obtained after subtraction of the control group Ct value from the Ct value from MCF-7 cells with 5-aza-dC treatment, indicate that this DNA region was more accessible, whereas negative values indicate the opposite. We found that the region from +1 to +400 bp was more open to nuclease digestion in MCF-7 cells with 5-aza-dC treatment compared to control group (Fig. 5b). This result suggests that 5-aza-dC might alter CLDN6 chromatin structure for its transcription in breast cancer cells.

### DNA demethylation restores CLDN6 expression, inhibiting invasion and migration in MCF-7 cells

**Fig. 6:**
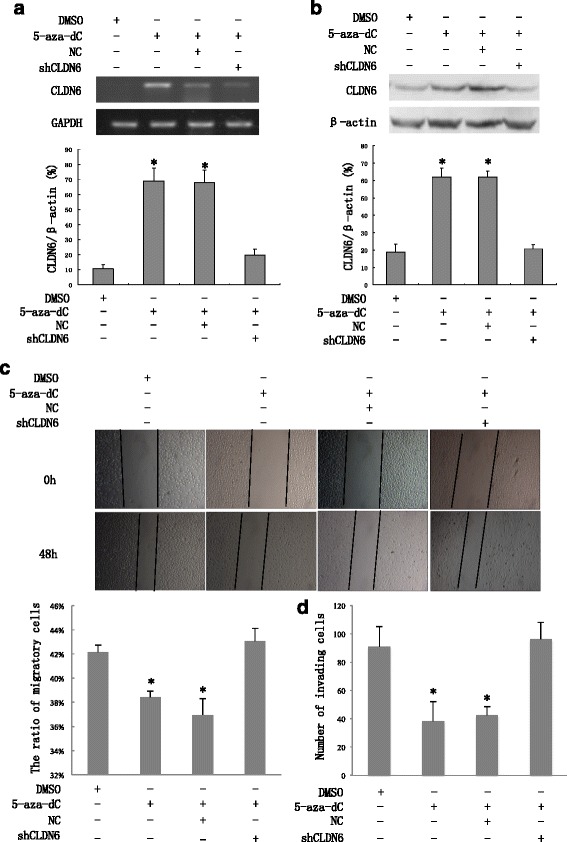
CLDN6 acts as a critical role in suppressing tumorigenesis in breast cancer cells. **a** MCF-7 cells were mock transfected or transfected with siRNA for CLDN6. Nontargeting siRNA was used as negative control. Total RNA was extracted 48 h after transfection, and CLDN6 mRNA levels were determined using quantitative RT-PCR. **b** Total protein was extracted 48 h after transfection, and CLDN6 expression at protein levels was determined using western blot assay. **c** Knockdown of CLDN6 in MCF-7 cells, the migratory abilities of treating with 5-aza-dC group and 5-aza-dC with vector group were significantly reduced than control and 5-aza-dC with CLDN6 RNAi group. **d** Knockdown of CLDN6 in MCF-7 cells, the invasive abilities of treating with 5-aza-dC group and 5-aza-dC with vector group were significantly reduced than control and 5-aza-dC with CLDN6 RNAi group. *P < 0.05. CLDN6, Claudin-6; 5-aza-dC, 5-Aza-2’-deoxycytidine

We knockdowned CLDN6 and confirmed its efficiency at mRNA and protein levels in MCF-7 cells treated with 5-aza-dC, (Fig. 6a and b) and found knockdown of CLDN6 restored migratory and invasive abilities (Fig. 6c and d).

All together, we demonstrated that DNA methylation down-regulated CLDN6 expression via recruiting MeCP2 and deacetylating H3Ac and H4Ac, indicating loss of CLDN6 expression could further destroy integrity of cells by losing cell polarity and cell adhesion, and promote malignant progression. It is interesting that DNA methyltransferase inhibitor 5-aza-dC and histone deacetylase inhibitor TSA could induce CLDN6 re-expression partially and 5-aza-dC could inhibit invasion and migration of MCF-7 cells. Overall, these data suggest that CLDN6 plays a critical role in suppressing malignant progression in MCF-7 cells.

## Discussion

In our previous work, we have identified CLDN6 as a potential mammary cancer suppressor gene, which may contribute to mammary cancer resistant phenotype observed in human and rat mammary cancer cell lines [5, 6]. However, its regulation mechanisms are elusive. Therefore, investigation of the mechanism of regulating CLDN6 expression may help in understanding of breast tumorigenesis and provide new opportunities for detection and therapy of this disease. Promoter hypermethylation, with MeCPs binding and histone deacetylation, has been identified as a major epigenetic event associated with the loss of tumor suppressor gene expression during cancer progression [23–25]. DNA methylation-dependent repression of transcription can be mediated by several mechanisms. At first, DNA methylation at binding sites of transcription factors can repress the transcription factor. A methylated target sequence can interfere transcription factors such as Sp1/Sp3, E2F and CREB to bind to their binding sites and to prevent transcriptional activation [17, 26]. A more elaborate mechanism is that methyl-CpG binding proteins such as MeCP2 and MBD2, a member of the MeCP1 complex [27], are recruited to the methyl-CpG sequence and then these methyl-CpG binding proteins are associated with various chromatin modifiers to establish a repressive chromatin environment [18, 27–29].

In this report, we examined CLDN6 expression in HBL-100, MCF-7 and COC1 cells by RT-PCR and western blot assay (Fig. 1b) and the methylation status at the CLDN6 gene CpG sites and found that MCF-7 cells did not express CLDN6 with methylation at the CLDN6 CpG sites. HBL-100 and COC1 cells with CLDN6 expression exhibited unmethylation at CLDN6 CpG sites. To determine the contribution of DNA methylation and histone modification in CLDN6 expression, we treated MCF-7 cells with 5-aza-dC and TSA. The CLDN6 expression was up-regulated by treating with 5-aza-dC and TSA respectively and synergistically, indicating that DNA methylation and histone deacetylation play an essential role in CLDN6 silence.

Our data indicate that the methylation of CpG dinucleotides in the CLDN6 promoter may not directly interfere with the binding ability of transcription factor, but methyl-CpG binding protein may interfere with it. However, the role of methyl-CpG-binding proteins in the decrease of transcription factor binding in the methylated CLDN6 promoter is not clear. Nevertheless, our ChIP assay suggested that MeCP2 did interact with CLDN6 proximal promoter in MCF-7 cells in vitro, but not in 5-aza-dC treatment group. These results demonstrated that methylated CLDN6 promoter interfered transcription of CLDN6, which was likely intermediated by MeCP2 protein.

Methyl-CpG binding proteins (MBPs) bind to regions of methylated DNA and form complexes with HDACs, then resulting in histone deacetylation downstream of the methylated CpG sequence and transcriptional silencing of that region of the genome [30]. Similarly, proteins that either bind modified histones such as heterochromatin protein 1 (HP1) or directly modify histones such as lysine specific demethylase 1 (LSD1) and protein arginine methyltransferase 5 (PRMT5), can recruit DNA methyltransferases to induce DNA methylation in the silenced gene [31–33]. Furthermore, our data showed TSA could up-regulate CLDN6 expression, indicating that not only DNA methylation, but also histone deacetylation play an essential role in CLDN6 silencing in MCF-7 cells. We determined H3Ac and H4Ac in CLDN6 promoter in MCF-7 cells with 20 μM 5-aza-dC and 125nM TSA treatment for 48 h respectively and simultaneously. H4Ac in CLDN6 promoter was induced by using 5-aza-dC and TSA respectively and simultaneously, but H3Ac in CLDN6 was induced only with 5-aza-dC treatment, suggesting that deacetylation of H3Ac might depend on DNA methylation to repress CLDN6 expression, but H4Ac might not rely on DNA methylation, and might independently regulate CLDN6 expression. Histone modification may repress transcriptional complexes to combine with gene promoter by changing the chromatin structure in nucleosomes and in turn making the complexes inaccessible to the transcriptional machinery. Our nucleosomal mapping experiment indicated that indeed the DNA methylation of CLDN6 promoter in MCF-7 cells existed in a more organized nucleosomal form that is more resistant to endonuclease digestion. Collectively, our results indicated that 5-aza-dC and TSA treatment in MCF-7 cells could increase the expression of CLDN6, and suggested that the deacetylase inhibitor TSA may partly relax demethylated chromatin and activate transcription.

Additional studies are needed to determine the exact molecular mechanism of DNA methylation, and whether there is a link between DNA methylation and histone deacetylation. Our study showed MeCP2 was a key protein that bound to methylated DNA regions and recruited HDACs, and formed complexes that leaded to histone deacetylation and silenced the transcriptional activity of that region of the genome [17, 28, 27]. Our ChIP assay experiments indicated that MeCP2 did interact with the CLDN6 promoter in MCF-7 cells in vitro, we presumed that methylated DNA might recruit MeCP2, which can recruit HDACs to repress CLDN6 expression. It is possible to have other potential recruited complexes for methylation.

## Conclusion

CLDN6 expression is related to DNA methylation in breast cancer tissues and MCF-7 cells. DNA methylation of CLDN6 gene down-regulates its expression by recruiting MeCP2, deacetylating H3 and H4, and altering chromatin structure. Down-regulation of CLDN6 expression could facilitate migratory and invasive phenotype in breast cancer cells.

## Abbreviations

CLDN6, Claudin-6; TJ, Tight junction; 5-aza-dC, 5-Aza-2’-deoxycytidine; TSA, Trichostatin A; MSP, Methylation-Specific PCR; EMT, Epithelial-mesenchymal transition; MeCP2, methyl-CpG-binding protein 2; JAM, Junctional adhesion molecules; mCpGs, methylated CpGs; HDAC, histone deacetylase; H3Ac, Histone 3 acetylation; H4Ac, Histone 4 acetylation; MBP, Methyl-CpG binding protein; HP1, Heterochromatin protein 1; LSD1, Lysine specific demethylase 1; PRMT5, Protein arginine methyltransferase 5.
